# Hybrid Nanostructured Porous Silicon-Silver Layers for Wideband Optical Absorption

**DOI:** 10.1038/s41598-019-43712-7

**Published:** 2019-05-13

**Authors:** Raúl J. Martín-Palma, Patrick D. McAtee, Rehab Ramadan, Akhlesh Lakhtakia

**Affiliations:** 1 Department of Materials Science and Engineering, The Pennsylvania State University, University Park, Pennsylvania, PA 16802 USA; 2 Departamento de Física Aplicada, Universidad Autónoma de Madrid, 28049 Cantoblanco, Madrid, Spain; 3 Department of Engineering Science and Mechanics, The Pennsylvania State University, University Park, Pennsylvania, PA 16802 USA; 4 Department of Physics, Faculty of Science, Minia University, 61519 Minia, Egypt

**Keywords:** Nanoscale materials, Materials for optics, Optical materials and structures, Condensed-matter physics

## Abstract

As subwavelength nanostructures are receiving increasing attention for photonic and plasmonic applications, we grew nanostructured porous silicon (n-PS) and hybrid n-PS/Ag layers onto silicon substrates and measured their reflection and absorption characteristics as functions of the wavelength, angle of incidence, and polarization state of incident light. The experimental results show that the absorption characteristics of the hybrid n-PS/Ag layer can be controlled by selecting the appropriate combination of its thickness and porosity, together with the density of infiltrant silver nanoparticles. The observed wideband optical absorption characteristics of the hybrid n-PS/Ag layers might be useful in light-harvesting devices and photodetectors, since the overall efficiency will be increased as a result of increased field-of-view for both *s*- and *p*-polarization states of incident light.

## Introduction

Although nanostructured porous silicon (n-PS) was discovered by Uhlir in 1956 while studying electropolishing of silicon and germanium in HF-based solutions^1^, it was not until the observation visible photoluminescence from room-temperature n-PS by Canham in 1990^2^ that this material was envisaged as a good candidate for applications in the field of photonics. Since then, its many additional properties beyond photo- and electro-luminescence have resulted in several successful applications in different fields ranging from microelectronics to biomedicine^3^.

Nanostructured porous silicon can be described quite simply as an amorphous matrix in which silicon nanocrystals are embedded^4–7^. The morphology of a n-PS sample depends on strongly on the specific fabrication parameters, including electrolyte composition and fabrication current density^8^. The complex-valued effective refractive index of n-PS is therefore dependent on the electrochemical fabrication parameters^9^. Control over the refractive index allows the use of n-PS for tunable photonic structures made entirely of silicon^10^. It is worth pointing out that the electrochemical technique used for the fabrication of n-PS is compatible with current CMOS fabrication processes, thus enabling the potential integration of n-PS into circuits using microelectronics-compatible procedures.

Within this context and given the increasing attention being received by subwavelength nanostructures for photonic and plasmonic applications^11,12^, we decided to explore the use of n-PS and hybrid n-PS/Ag layers grown onto silicon as wideband optical absorbers. Therefore, we experimentally investigated the dependence of optical absorption characteristics of n-PS and hybrid n-PS/Ag layers on the thickness and porosity of n-PS layers and the amount of silver infiltrating the pores of n-PS. More specifically, we measured the reflection and absorption characteristics of the n-PS and hybrid n-PS/Ag layers as functions of the wavelength, angle of incidence, and polarization state of incident light.

## Experimental

### Fabrication of n-PS and hybrid n-PS/Ag layers

**Figure 1:**
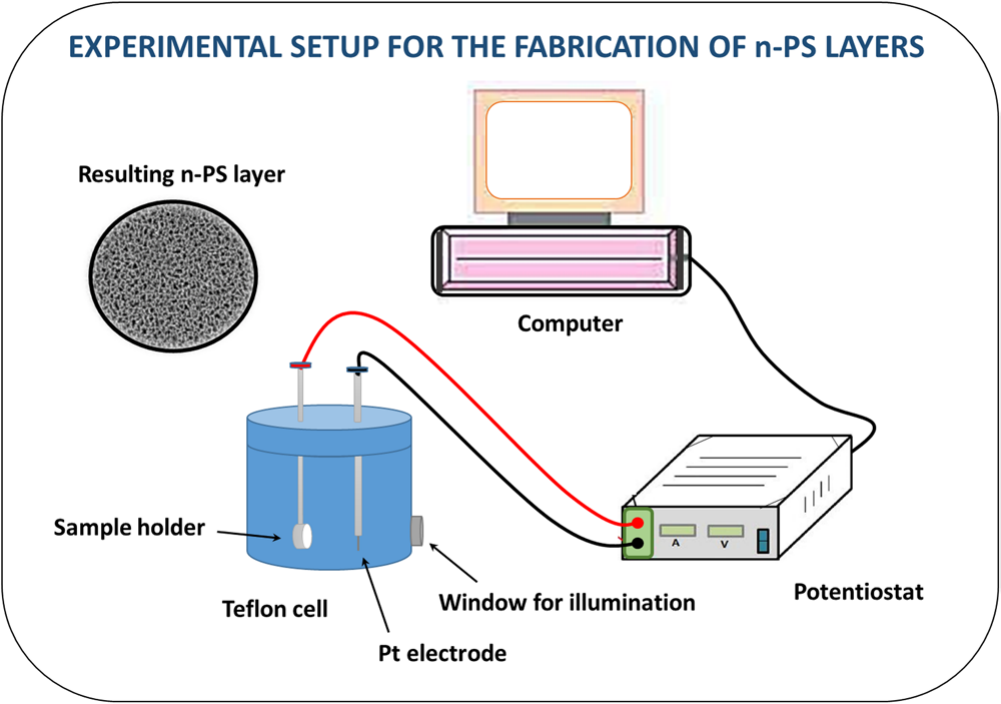
Experimental setup used for the fabrication of n-PS layers by the electrochemical etch of silicon wafers.

Nanostructured porous silicon layers were formed by the electrochemical etching of boron-doped (*p*-type) silicon wafers of <100> orientation, resistivity in the 0.01–0.02 Ω·cm range, and surface roughness on the order of 0.1 nm. The wafers were cut into 1.5 × 1.5 cm^2^ pieces. Each piece was mounted in a sample holder with about 1.23 cm^2^ area exposed to the etching solution, which was formulated as a 1:2 HF (48 wt %):ethanol (98 wt %) mixture. The anodization times, ranging from 18–46 s, were adjusted to obtain layers of three different thickness. Two different current densities — namely, 20 and 60 mA/cm^2^ — were used, leading to two different porosities arising from the creation of nanocolumnar pores of two different average diameters. After fabrication, each sample was rinsed in ethanol and blown dry with a mild stream of dry nitrogen. The experimental setup used for the fabrication of n-PS layers by the electrochemical etch of silicon wafers is schematically depicted in Fig. 1.

The n-PS layer was used as a template for the subsequent growth of silver nanoparticles inside the porous structure. For this task, a modified electrochemical deposition process^13^ was implemented. This process was carried out using a Bio-Logic SP-150 potentiostat at a fixed current density of 1 μA/cm^2^ for different electrodeposition durations (2, 4, 6 or 15 min) in an aqueous solution of silver nitrate (0.1 mM), sodium citrate (0.25 mM), and nitric acid (0.01 M), with pH = 3. Afterwards, the sample was rinsed in ethanol for 5 min and subsequently distilled in water for another 5 min and finally dried with a mild stream of dry nitrogen. This process leads to the formation of a hybrid n-PS/Ag thin film.

In all, four different sets of n-PS and hybrid n-PS/Ag layers were prepared as detailed in Table 1, to study the effects of porosity, thickness, and the volumetric proportion of silver on the optical response characteristics.

**Table 1:** Summary of the fabrication conditions of the n-PS layers and hybrid n-PS/Ag layers.

Current density (mA/cm^2^)	Duration of anodization (s)	Thickness (µm)	Duration of electrodeposition (min)
n-PS and hybrid n-PS/Ag layers			
20 (low porosity)	22	0.33	0
	33	0.60	0
	46	0.77	0
60 (high porosity)	18	0.70	0
	28	1.00	0
	40	1.40	0
60 (high porosity)	18	0.70	2, 4, 6, or 15
	28	1.00	2, 4, 6, or 15
	40	1.40	2, 4, 6, or 15

### Morphological characterization

Top-view and cross-sectional images of every sample were acquired using a Philips XL 30S-FEG field emission scanning electron microscope (FESEM). The sizes of the pores of n-PS and the Ag nanoparticles were determined from these FESEM images using the ImageJ software.

### Optical characterization

#### Specular reflection

White light from a halogen source (HL-2000, Ocean Optics, Dunedin, Florida, USA) was passed through a fiber-optic cable and then through a linear polarizer (GT10, ThorLabs, Newton, NJ, USA) to be incident upon the sample to be characterized. The reflected light was passed through a second linear polarizer (GT10, ThorLabs) and then through a fiber-optic cable to a CCD spectrometer (HRS-BD1-025, Mightex Systems, Pleasanton, CA, USA). All measurements were taken in a dark room and background noise was removed from the measured data. Co-polarized specular reflectances (*R*_*ss*_ and *R*_*pp*_) and cross-polarized specular reflectances (*R*_*ps*_ and *R*_*sp*_) were measured for the angle of incidence *θ*_*inc*_ in the 10°−70° range as functions of the free-space wavelength *λ*_*o*_ in the 400–900 nm visible and near-infrared spectral regimes. The angle of incidence is measured with respect to the perpendicular to the illuminated face of the sample, with *θ*_*inc*_ = 0° for normal incidence. The first subscript on a specular reflectance denotes the linear polarization state of light collected by the detector, while the second subscript denotes the linear polarization state of light impinging on the sample. Care was taken to remove the effects of ambient light, as described elsewhere^14^.

#### Overall reflection

The overall reflectance spectrum for *λ*_*o*_ in the 250–900 nm wavelength regime was acquired using a double-beam spectrophotometer (V-560, JASCO International, Tokyo, Japan) equipped with an integrating sphere in order to collect both diffuse and specular reflections, with unpolarized light being incident at angle *θ*_*inc*_ = 10° (i.e., almost normally) on the sample. The photometric accuracy is specified by the manufacturer to be better than 0.3%.

## Experimental Results

**Figure 2:**
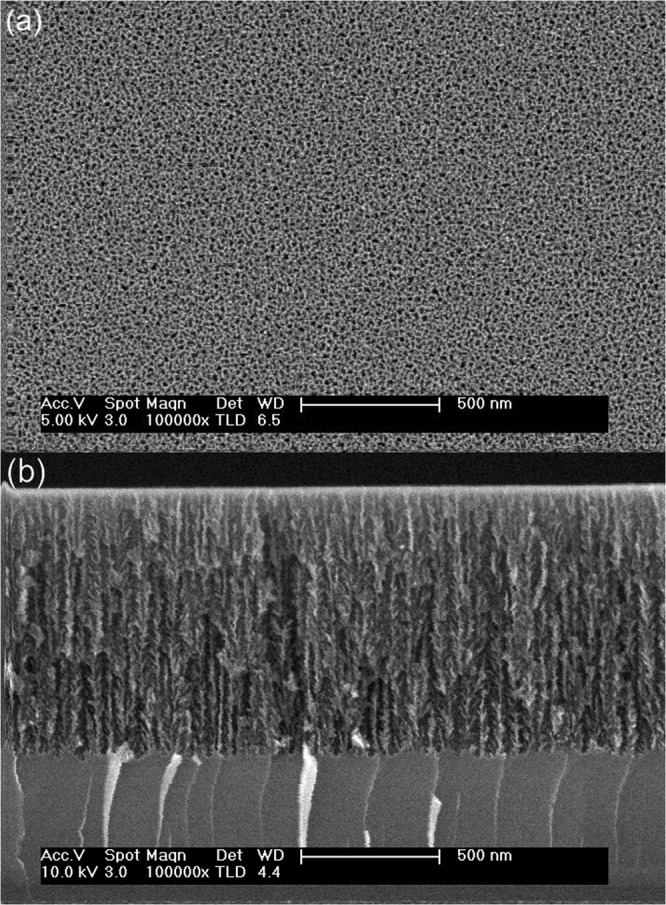
(**a**) Top-view and (**b**) cross-sectional FESEM images of a typical high-porosity n-PS layer (fabrication current density of 60 mA/cm^2^).

Figure 2 shows top-view and cross-sectional FESEM images of a typical n-PS layer grown by the electrochemical etch of a monocrystalline silicon wafer. Clearly, the electrochemical fabrication process delivers a porous columnar structure. For low-porosity n-PS layers (fabrication current density of 20 mA/cm^2^), the pore diameter is in the 5–15 nm range, as determined from FESEM image analysis, but that range changes to 15–35 nm in the case of high-porosity n-PS layers (60 mA/cm^2^).

**Figure 3:**
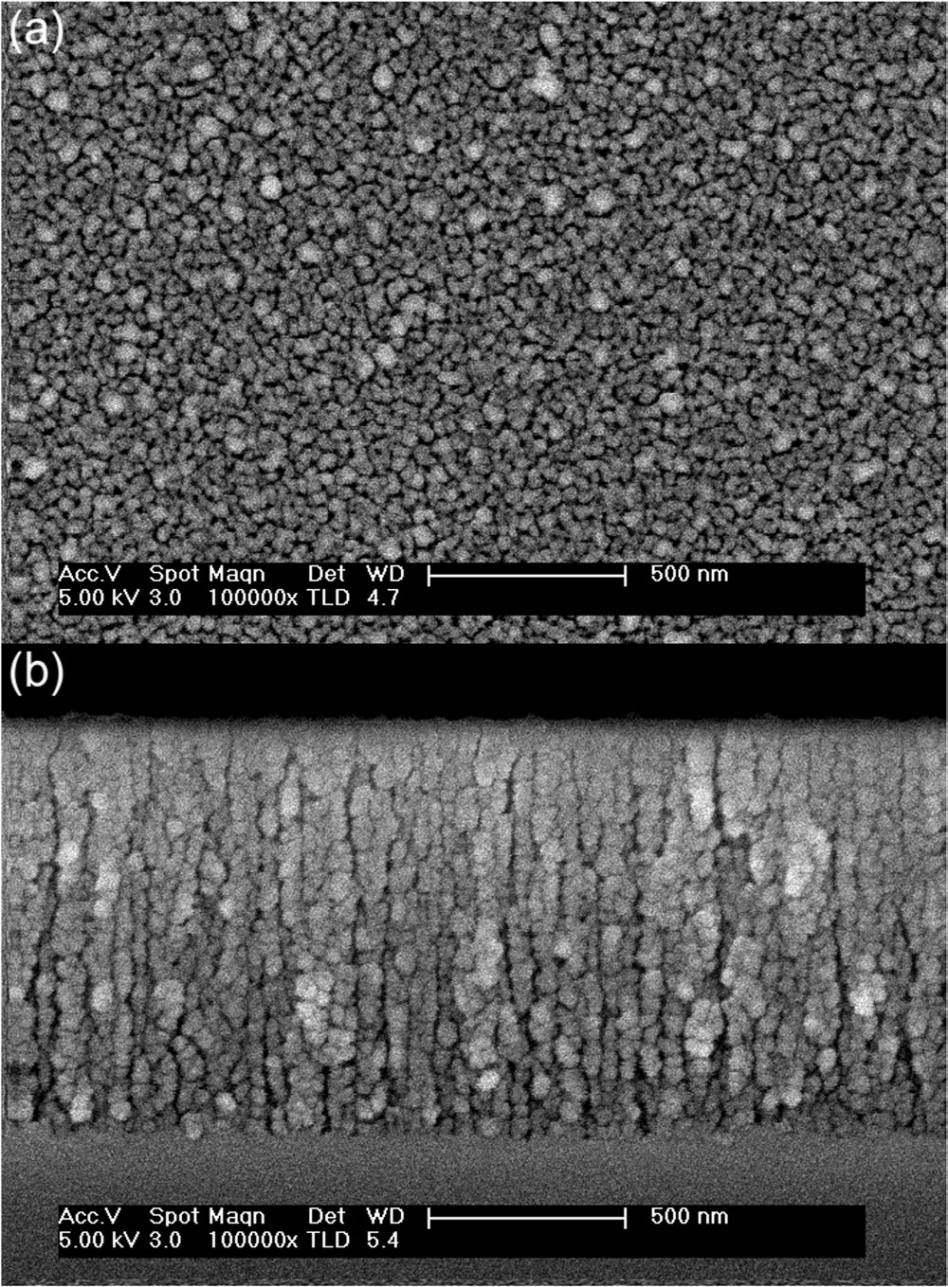
Top-view and cross-sectional FESEM images of a typical hybrid n-PS/Ag layer. The n-PS layer was grown using a current density of 60 mA/cm^2^ and the silver nanoparticles were electrodeposited for 4 min. The Ag nanoparticles (bright areas) are rounded with diameters in the 30–65 nm range.

In Fig. 3, top-view and cross-sectional FESEM images of a typical hybrid n-PS/Ag layer grown onto silicon are presented. The sample comprises a high-porosity n-PS layer (current density 60 mA/cm^2^) in which silver nanoparticles were infiltrated for 4 min. The FESEM images show that the fabrication process leads to a quite homogeneous distribution of silver nanoparticles on the surface and inside the n-PS layer, the nanoparticle diameter ranging from 30–65 nm as determined by image analysis. Accordingly, given their characteristic small feature sizes, both the n-PS and the hybrid n-PS/Ag layers can be treated as homogeneous media from an optical standpoint at any wavelength *λ*_*o*_ exceeding about 200 nm.

**Figure 4:**
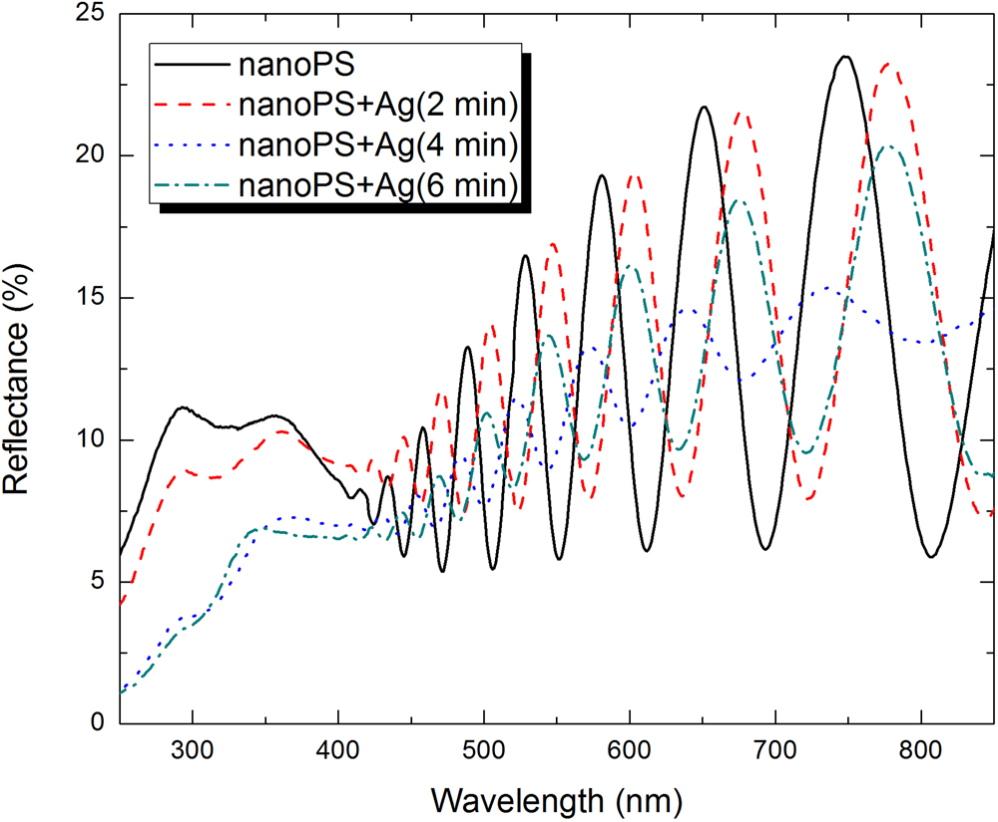
Overall reflectance spectra of 1.4-µm-thick high-porosity n-PS and hybrid n-PS/Ag layers for different durations of infiltration of silver nanoparticles by electrodeposition (2, 4, and 6 min).

Aiming at increasing optical absorption in the hybrid n-PS/Ag layers, we controlled the volumetric fraction of silver nanoparticles by using three different electrodeposition durations: namely, 2, 4, and 6 min. From the overall reflectance spectra shown in Fig. 4, we concluded that the differences between neighboring maxima and minima are the least when the electrodeposition duration was 4 min.

Reflection reduction after electrodeposition for 2 min can be attributed to increased absorption due to a strong interaction between free electrons in the silver nanoparticles and the incident electromagnetic radiation^15^. Reflection reduction is enhanced after electrodeposition for 4 min, as a consequence of increased absorption due to a larger number density of silver nanoparticles. However, increasing the electrodeposition duration to 6 min results in increased reflection, because the even larger number density of silver nanoparticles leads to the percolation effect^16,17^, the hybrid n-PS/Ag layer acquiring a strongly metallic character that inhibits refraction into the layer.

**Figure 5:**
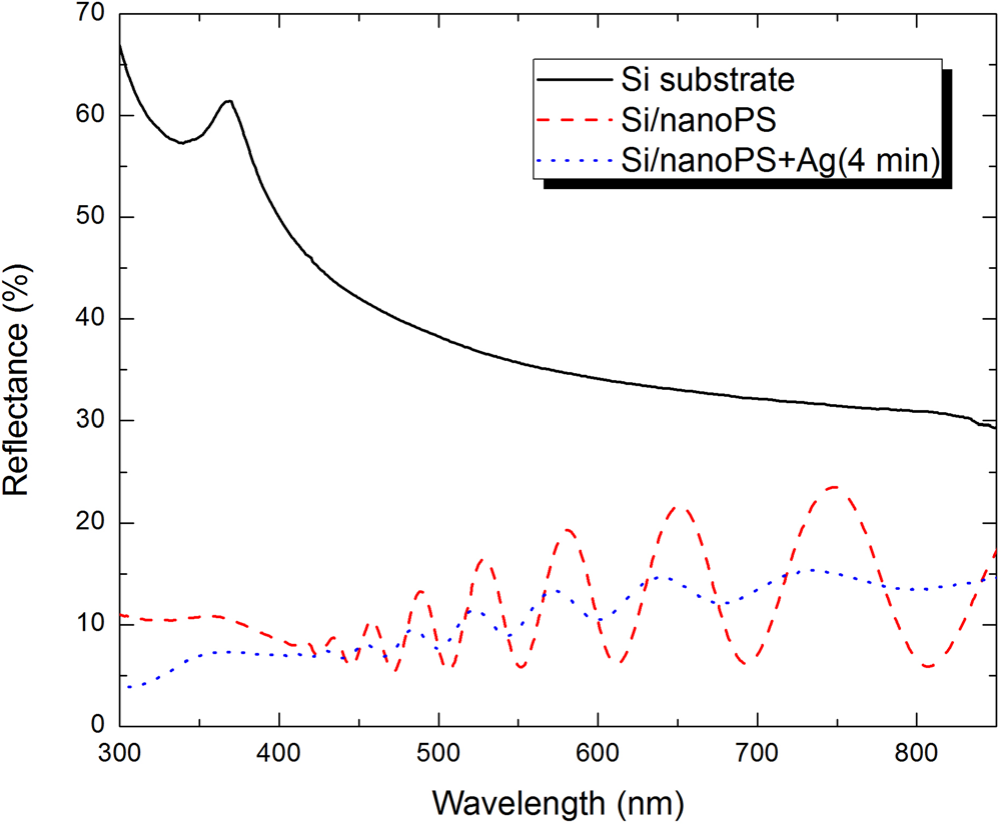
Overall reflectance spectra of bulk silicon substrate, a 1.4-µm-thick high-porosity n-PS layer on a silicon substrate, and a 1.4-µm-thick high-porosity n-PS layer (on a silicon susbtrate) infiltrated by silver nanoparticles during 4-min electrodeposition.

The overall reflectance spectra of the high-porosity n-PS layers and the best hybrid n-PS/Ag layers are shown in Fig. 5 in addition to the overall reflectance spectrum of a silicon wafer. It is evident that the fabrication of n-PS onto silicon substrates results in a remarkable reduction of the average overall reflectance and that further reflection reduction occurs on infiltration by silver nanoparticles. This effect is attributable to increased optical absorption provided by the silver nanoparticles in consequence of the high values of the imaginary part of the refractive index of silver in the chosen spectral regime^18–20^. Additionally, the observed shifts in the reflectance maxima and minima can be attributed to the changes in the spectrum of the complex-valued effective refractive index as a consequence of: first, due to the creation of pores due to electrochemical etching and, second, to the electrodeposition of silver nanoparticles.

**Figure 6:**
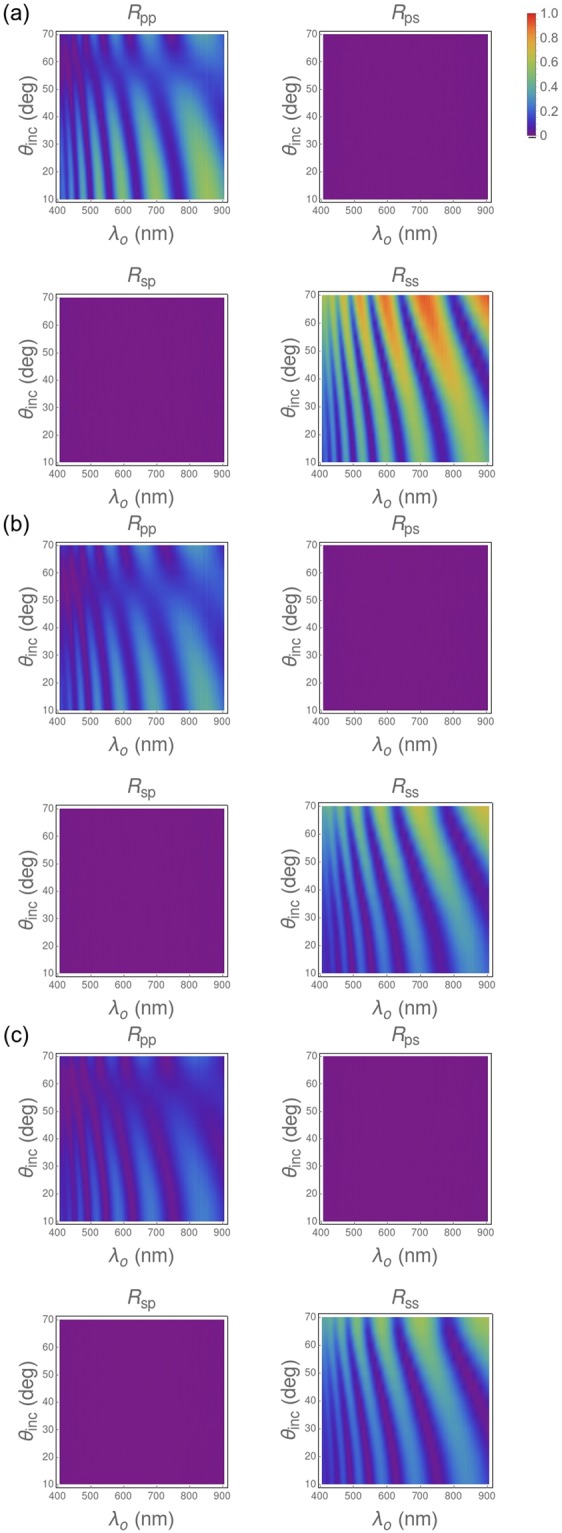
Dependences on *λ*_*o*_ and *θ*_*inc*_ of the measured specular reflectances of (**a**) a 1-µm-thick high-porosity n-PS layer, (**b**) a 1-µm-thick high-porosity hybrid n-PS/Ag layer after silver infiltration for 15 min, and (**c**) a 1-µm-thick high-porosity hybrid n-PS/Ag layer after silver infiltration for 4 min.

Figure 6 shows characteristic two-dimensional density plots of co-polarized and cross-polarized specular reflectances as functions of *λ*_*o*_ and *θ*_*inc*_ for a n-PS layer and two hybrid n-PS/Ag layers grown onto monocrystalline substrates. First, it is worth noticing that the two cross-polarized reflectances (*R*_*ps*_ and *R*_*sp*_) are negligible for all n-PS layers and hybrid n-PS/Ag layers, independently of their porosity and thickness. Therefore, materials of both types can be considered to be isotropic in the plane normal to the thickness direction. Second, we found that the reflectance spectra did not change when the sample was translated transversely to the path of the incident light beam. Therefore, materials of both types can be considered to be effectively homogeneous, as also indicated by the FESEM images.

The reflectance spectra in Fig. 6 contain several bands having their origin in classical interference effects. The bright bands (or fringes) correspond to interference maxima while the dark bands correspond to interference minima. We ascertained from the reflectance spectra of all 18 samples mentioned in Table 1 that the number of bright bands increases with the sample thickness. Also, there is an evident dependence of the width of the bright bands on the angle of incidence, which is a consequence of increased optical path for larger angles of incidence.

In the plots of *R*_*pp*_ in Fig. 6, we see a dark horizontal region corresponding to low reflection in the vicinity of *θ*_*inc*_ = 60°, but a similar dark region is absent in the plots of *R*_*ss*_. This is indicative of the pseudo-Brewster effect^21^. As *θ*_*inc*_ increases further, both *R*_*pp*_ and *R*_*ss*_ increase rapidly towards unity.

**Figure 7:**
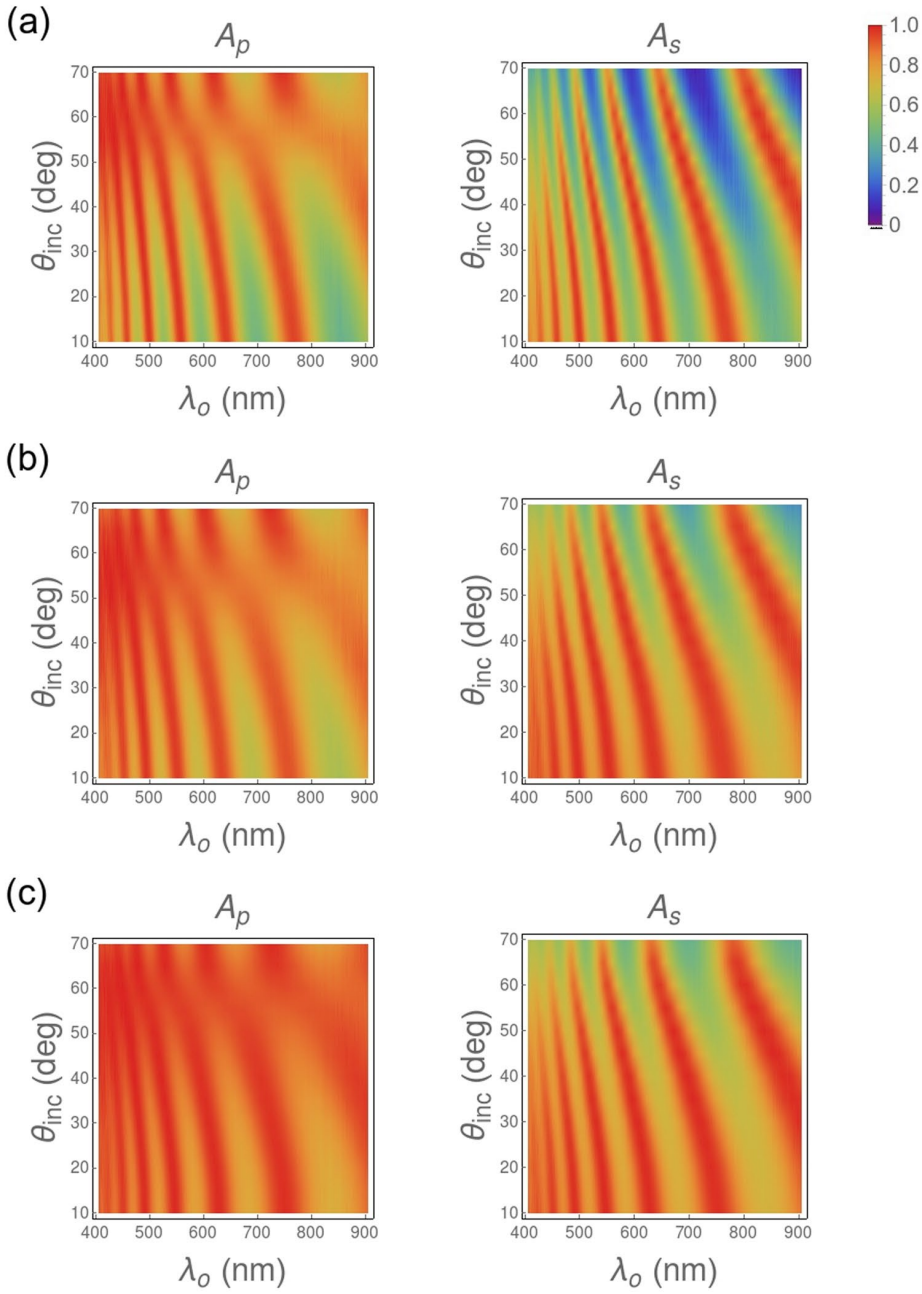
Dependences on *λ*_*o*_ and *θ*_*inc*_ of the absorptances of (**a**) a 1-µm-thick high-porosity n-PS layer, (**b**) a 1-µm-thick high-porosity hybrid n-PS/Ag layer after silver infiltration for 15 min, and (**c**) a 1-µm-thick high-porosity hybrid n-PS/Ag layer after silver infiltration for 4 min.

We ascertained experimentally that no light was transmitted through any of the samples. Therefore, we defined the absorptances *A*_*p*_ = 1− (*R*_*pp*_ + *R*_*sp*_) and *A*_*s*_ = 1− (*R*_*ss*_ + *R*_*ps*_) and plotted them as functions of *λ*_*o*_ and *θ*_*inc*_ in Fig. 7 for a n-PS layer and two hybrid n-PS/Ag layers grown onto silicon substrates. Clearly in this figure, *A*_*p*_ is larger than *A*_*s*_ for all three samples. Besides, the comparison of Fig. 7a,b allows the conclusion that infiltration of n-PS layers by silver nanoparticles results in increased absorption, given that broader and more intense absorption bands are displayed. This enhancement is observed for both *A*_*p*_ and *A*_*s*_, although it is more pronounced for *A*_*s*_. Absorption is further increased once the density of silver particles in the nanoporous structure is optimized, as shown in Fig. 7c. Accordingly, it can be concluded that the infiltration of n-PS by silver nanoparticles results in increased absorption and, additionally, optical absorption can be controlled by controlling the density of the infiltrant silver nanoparticles.

## Concluding Remarks

The use of nanostructured porous silicon (n-PS) and hybrid n-PS/Ag layers grown onto silicon as wideband optical absorbers with potential applications in the fields of light sensing and light harvesting was experimentally explored by investigating the dependences of the optical reflectances and absorptances on the thickness and porosity of n-PS and hybrid n-PS/Ag layers and the number density of the infiltrant silver nanoparticles.

Our data show that the absorption characteristics of the hybrid n-PS/Ag layers can be controlled by selecting the appropriate combination of thickness and porosity of the n-PS layers, together with the density of infiltrant silver nanoparticles. The wideband optical absorption characteristics of the hybrid n-PS/Ag layers are expected to contribute to increased efficiency of light-harvesting devices and photodetectors given by increased field-of-view for both *s*- and *p*-polarization states of incident light over a broad spectral regime.
